# Diabetes Minimally Mediated the Association Between PM_2.5_ Air Pollution and Kidney Outcomes

**DOI:** 10.1038/s41598-020-61115-x

**Published:** 2020-03-12

**Authors:** Benjamin Bowe, Yan Xie, Yan Yan, Hong Xian, Ziyad Al-Aly

**Affiliations:** 1 Clinical Epidemiology Center, Research and Education Service, VA Saint Louis Health Care System, Saint Louis, Missouri USA; 2 Department of Epidemiology and Biostatistics, College for Public Health and Social Justice, Saint Louis University, Saint Louis, Missouri USA; 3 Veterans Research & Education Foundation of St. Louis, Saint Louis, Missouri USA; 4 Division of Public Health Sciences, Department of Surgery, Washington University School of Medicine, Saint Louis, Missouri USA; 5 Department of Medicine, Washington University School of Medicine, Saint Louis, Missouri USA; 6 Nephrology Section, Medicine Service, VA Saint Louis Health Care System, Saint Louis, Missouri USA; 7 Institute for Public Health, Washington University in Saint Louis, Saint Louis, Missouri USA

**Keywords:** Environmental impact, Diabetes, Chronic kidney disease

## Abstract

Epidemiologic observations suggest that exposure to ambient fine particulate matter (PM_2.5_) is associated with increased risk of chronic kidney disease (CKD) and diabetes, a causal driver of CKD. We evaluated whether diabetes mediates the association between PM_2.5_ and CKD. A cohort of 2,444,157 United States veterans were followed over a median 8.5 years. Environmental Protection Agency data provided PM_2.5_ exposure levels_._ Regression models assessed associations and their proportion mediated. A 10 µg/m^3^ increase in PM_2.5_ was associated with increased odds of having a diabetes diagnosis (odds ratio: 1.18, 95% CI: 1.06–1.32), use of diabetes medication (1.22, 1.07–1.39), and increased risk of incident eGFR <60 ml/min/1.73 m^2^ (hazard ratio:1.20, 95% CI: 1.13–1.29), incident CKD (1.28, 1.18–1.39), ≥30% decline in eGFR (1.23, 1.15–1.33), and end-stage renal disease (ESRD) or ≥50% decline in eGFR (1.17, 1.05–1.30). Diabetes mediated 4.7% (4.3–5.7%) of the association of PM_2.5_ with incident eGFR <60 ml/min/1.73 m^2^, 4.8% (4.2–5.8%) with incident CKD, 5.8% (5.0–7.0%) with ≥30% decline in eGFR, and 17.0% (13.1–20.4%) with ESRD or ≥50% decline in eGFR. Diabetes minimally mediated the association between PM_2.5_ and kidney outcomes. The findings will help inform more accurate estimates of the burden of diabetes and burden of kidney disease attributable to PM_2.5_ pollution.

## Introduction

Experimental studies and epidemiologic observations suggest that exposure to higher levels of air pollution, specifically ambient particulate matter less than 2.5 µm in diameter (PM_2.5_), is associated with increased risk of incident chronic kidney disease (CKD), CKD progression, and end stage renal disease (ESRD)^1–3^. A large body of evidence — both mechanistic and epidemiologic research — also suggests that exposure to higher levels environmental air pollution, and in particular PM_2.5_, is also associated with increased risk of diabetes — a causal driver of CKD^4^. However, whether the described association between PM_2.5_ and risk of kidney disease is mediated in part or fully by diabetes is not known. Addressing this knowledge gap will help a) enhance our understanding of how exposure to fine particulate matter air pollution affects kidney function, and b) inform more accurate estimates of the burden of kidney disease and burden of diabetes attributable to PM_2.5_ pollution^5,6^. In this work we aimed to address this knowledge gap and built a cohort of United States veterans to estimate the proportion of the association between PM_2.5_ and adverse kidney outcomes which is mediated by diabetes.

## Results

**Figure 1:**
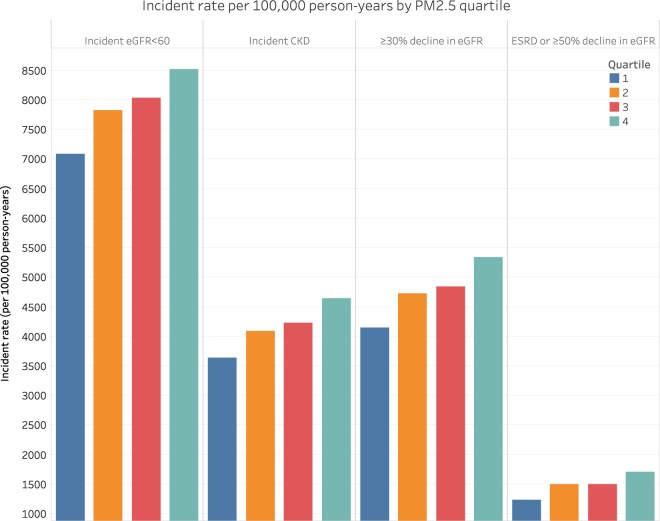
Adjusted incident rates of adverse kidney disease outcomes by PM_2.5_ quartile. Rates adjusted for age, race, sex, and T_0_ eGFR.

A cohort of 2,444,157 United States veterans were followed over a median 8.5 years (IQR: 8.0–8.8). The geographic distribution of cohort participants is mapped in Supplementary Figure S1. Demographic and health characteristics of the overall cohort and by PM_2.5_ quartile are provided in Table 1. Compared to the lowest quartile of PM_2.5_, a higher proportion of those in the highest quartile of PM_2.5_ were black, were diagnosed with diabetes or were taking a medication for diabetes, and had a higher T_0_ estimated glomerular filtration rate (eGFR). Adjusted incident rates of kidney disease outcomes increased across increasing PM_2.5_ quartiles (Fig. 1, Supplementary Table S1).

**Table 1:** Demographic and health characteristics of the overall study cohort and according to quartiles of annual average PM_2.5_ concentrations.

Characteristic	Overall Cohort	PM_2.5_ Quartile 1 5.0–10.1 μg/m^3^	PM_2.5_ Quartile 2 10.2–11.8 μg/m^3^	PM_2.5_ Quartile 3 11.9–13.7 μg/m^3^	PM_2.5_ Quartile 4 13.8–22.1 μg/m^3^
Number of Counties	3108	1175 (37.8)	769 (24.7)	810 (26.1)	354 (11.4)
Number of Cohort Participants (%)	2444157	615401 (25.2)	621458 (25.4)	511510 (25.0)	595788 (24.4)
Median Age (IQR)	62.5 (54.7–71.8)	63.3 (55.4–72.0)	62.7 (54.9–71.7)	61.9 (54.3–71.6)	62.1 (54.2–71.8)
Race (%)					
White	2005446 (82.1)	546695 (88.8)	538171 (86.6)	484062 (79.2)	436518 (73.3)
Black	356566 (14.6)	36270 (5.9)	64749 (10.4)	117235 (19.2)	138312 (23.2)
Other	82145 (3.4)	32436 (5.3)	18538 (3.0)	10213 (1.7)	20958 (3.5)
Gender (Male) (%)	2326872 (95.2)	586078 (95.2)	590412 (95.0)	581864 (95.2)	568518 (95.4)
Cancer (%)	286171 (11.7)	71593 (11.6)	72120 (11.6)	69742 (11.4)	72716 (12.2)
Cardiovascular Disease (%)	733819 (30.0)	178604 (29.0)	187514 (30.2)	188121 (30.8)	179580 (30.1)
Chronic Lung Disease (%)	479183 (19.6)	125096 (20.3)	126904 (20.4)	119060 (19.5)	108123 (18.2)
Diabetes Mellitus (%)					
Medication	532180 (21.8)	125122 (20.3)	132615 (21.3)	137437 (22.5)	137006 (23.0)
ICD-9 but no medication	155932 (6.4)	37262 (6.1)	39073 (6.3)	39494 (6.5)	40103 (6.7)
No diabetes	1756045 (71.9)	453017 (73.6)	449770 (72.4)	434579 (71.1)	418679 (70.3)
Hyperlipidemia (%)	1399687 (57.3)	354944 (57.7)	362716 (58.4)	351484 (57.5)	330543 (55.5)
Median Systolic Blood Pressure (IQR) (mmHg)	135.5 (125.7–145.5)	135.0 (125.3–144.7)	136.0 (126.0–145.6)	135.5 (125.5–145.8)	135.6 (125.5–146.0)
Median Diastolic Pressure (IQR) (mmHg)	76.5 (70.0–82.8)	76.7 (70.3–82.8)	76.6 (70.2–82.7)	76.5 (70.0–82.8)	76.3 (70.0–82.8)
Peripheral Artery Disease (%)	66197 (2.7)	16781 (2.7)	16112 (2.6)	16890 (2.8)	16414 (2.8)
Smoking Status (%)					
Current	623226 (25.5)	142046 (23.1)	160416 (25.8)	161250 (26.4)	159514 (26.8)
Former	515859 (21.1)	123940 (20.1)	131294 (21.1)	125741 (20.6)	134884 (22.6)
Never	1305072 (53.4)	349415 (56.8)	329748 (53.1)	324519 (53.1)	301390 (50.6)
Body Mass Index (kg/m^2^)	28.7 (25.6–32.4)	28.7 (25.7–32.4)	28.8 (25.7–32.5)	28.7 (25.6–32.5)	28.6 (25.5–32.4)
ACEI/ARB use (%)	1153116 (47.2)	285477 (46.4)	293173 (47.2)	291925 (47.7)	282541 (47.4)
EPA Median County Particulate Matter 2.5 (IQR) (μg/m^3^)	11.8 (10.1–13.7)	9.1 (8.2–9.8)	11.1 (10.7–11.4)	12.7 (12.3–13.2)	15.1 (14.4–16.4)
NASA^+^ Median County Particulate Matter 2.5 (IQR) (μg/m^3^)	10.3 (7.7–12.9)	7.2 (5.8–8.5)	9.4 (7.6–10.8)	12.1 (10.7–13.3)	13.5 (11.4–14.9)
Median Air Sodium* (IQR) (μg/m^3^)	0.05 (0.04–0.08)	0.04 (0.03–0.08)	0.06 (0.04-0.11)	0.05 (0.04–0.08)	0.05 (0.04–0.08)
Median Follow-up Time (IQR) (years)	8.5 (8.0–8.8)	8.5 (8.1–8.8)	8.5 (8.0–8.8)	8.5 (8.0–8.8)	8.5 (8.0–8.8)
Death During Follow-up (%)	610215 (25.0)	149499 (24.3)	154857 (24.9)	152923 (25.0)	152936 (25.7)
Average eGFR at T_0_ (SD) (ml/min/1.73 m^2^)	76.2 (19.9)	74.5 (18.7)	76.0 (19.5)	76.2 (20.1)	78.2 (21.1)
Median Number of Outpatient eGFR Measures Before T_0_ (IQR)	4 (2–8)	5 (2–8)	4 (2–8)	4 (2–8)	4 (2–7)
With 1 or More Hospitalizations (%)	407447 (16.7)	97991 (15.9)	101020 (16.3)	103673 (17.0)	104763 (17.6)
Mean County Percent in Poverty (%) (SD)	13.3 (4.4)	12.8 (4.1)	13.3 (4.1)	13.9 (4.9)	13.4 (4.3)
Median Population Density (IQR) (per square mile)	249.9 (73.7–931.4)	81.6 (26.9–417.4)	183.1 (62.8–434.6)	284.5 (89.2–1115.3)	732.9 (266.7–2344.2)

Covariates as measured at T_0_. Abbreviations: PM_2.5_, particulate matter <2.5 µm in aerodynamic diameter; N, sample size; IQR, inter-quartile range; ACEI, angiotensin-converting enzyme inhibitors; ARB, angiotensin receptor blockers; SD, standard deviation; eGFR, estimated glomerular filtration rate; CI, 95% confidence interval; ESRD, end stage renal disease.

^+^In a subcohort with NASA estimated measures of PM_2.5_ (n = 2,361,129).

*In a subcohort within 30 miles of an air monitoring station that measures sodium (n = 1,164,532).

To establish the potential role of diabetes as a mediator in the association of PM_2.5_ with kidney disease outcomes, we first tested the association of PM_2.5_ and diabetes; our results suggest that a 10 μg/m^3^ increase in PM_2.5_ was associated with increased odds of diabetes (Table 2). PM_2.5_ was also associated with increased risk of kidney disease outcomes (Table 2). We additionally verified the presence and magnitude of the a priori established association of diabetes and risk of kidney disease (Table 2). Thus, diabetes may be a mediator in the relationship of PM_2.5_ and kidney disease outcomes (Table 2).

**Table 2:** Associations of ambient fine particulate matter (PM_2.5_) with diabetes and kidney disease outcomes, and of diabetes and kidney disease outcomes.

**The association of ambient fine particulate matter (PM**_**2.5**_**) with diabetes**					
**Exposure**		Diabetes*			
		**ICD-9 only** **OR (CI)**		Medication** **OR (CI)**	
**PM**_**2.5**_^**+**^		1.18 (1.06-1.32)		1.22 (1.07-1.39)	
**The association of ambient fine particulate matter** **(PM**_**2.5**_**) with kidney disease outcomes**					
**Exposure**		**Incident eGFR Less Than 60 ml/min/1.73 m**^**2**^ **HR (CI)**	**Incident CKD** **HR (CI)**	**≥30% Decline in eGFR** **HR (CI)**	**ESRD or ≥50% Decline in eGFR** **HR (CI)**
**PM**_**2.5**_^**+**^		1.20 (1.13-1.29)	1.28 (1.18-1.39)	1.23 (1.15-1.33)	1.17 (1.05-1.30)
**The association of diabetes with kidney disease outcomes**					
**Exposure**		**Incident eGFR Less Than 60 ml/min/1.73 m**^**2**^ **HR (CI)**	**Incident CKD** **HR (CI)**	**≥30% Decline in eGFR** **HR (CI)**	**ESRD or ≥50% Decline in eGFR** **HR (CI)**
Diabetes*	**ICD-9 only**	1.21 (1.20-1.23)	1.31 (1.29-1.33)	1.26 (1.24-1.27)	1.41 (1.38-1.44)
	Medication**	1.58 (1.56-1.59)	1.84 (1.82-1.86)	1.68 (1.67-1.70)	2.06 (2.02-2.10)
**Proportion of the association between PM2.5 and kidney disease mediated by diabetes**					
**Exposure**		**Incident eGFR Less Than 60 ml/min/1.73 m**^**2**^ **% (CI)**	**Incident CKD** **% (CI)**	**≥30% Decline in eGFR** **% (CI)**	**ESRD or ≥50% Decline in eGFR** **% (CI)**
**PM**_**2.5**_		4.7 (4.3-5.7)	4.8 (4.2-5.8)	5.8 (5.0-7.0)	17.0 (13.1-20.4)

Models adjusted for age, race, gender, cancer, cardiovascular disease, chronic lung disease, hyperlipidemia, T_0_ eGFR, body mass index, smoking status, ACEI/ARB use, systolic blood pressure, diastolic blood pressure, number of outpatient eGFR measurements, number of hospitalizations, county population density, and county percent in poverty.

^+^For every 10 μg/m^3^ increase in PM_2.5_

*No diabetes served as the reference category.

**Defined by intake of oral hypoglycemic agents or insulin.

Abbreviations: PM_2.5_, ambient particulate matter <2.5 µm in aerodynamic diameter; eGFR, estimated glomerular filtration rate; CKD, Chronic Kidney Disease; ESRD, end stage renal disease; CI, 95% confidence interval.

In order to resolve concerns about spurious associations and biases around the relationship of PM_2.5_ with diabetes and kidney disease outcomes, we used ambient air sodium levels, an exposure contextually similar to PM_2.5_, as a negative control^7^; there was an insignificant or vanishingly weak association between sodium and diabetes, and sodium and kidney disease outcomes (Supplementary Table S2).

**Figure 2:**
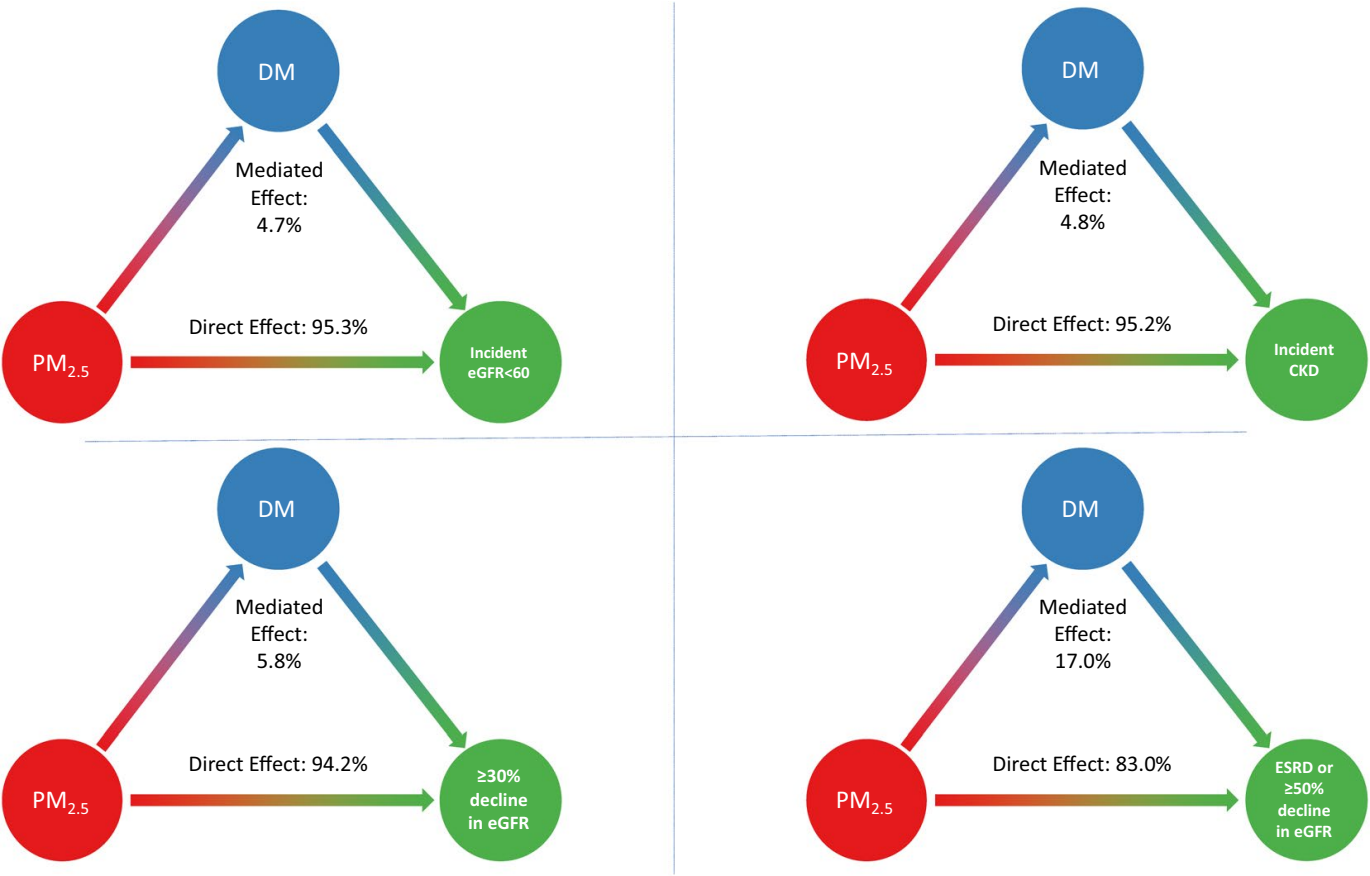
Proportion of the association between PM_2.5_ and kidney disease outcomes mediated by diabetes.

We then conducted mediation analyses to estimate the proportion of the association between PM_2.5_ and kidney disease outcomes that is mediated by diabetes. The proportion of the association mediated by diabetes was 4.7% (95%CI: 4.3–5.7%), 4.8% (4.2–5.8%), 5.8% (5.0–7.0%), and 17.0% (13.1–20.4%) for incident eGFR <60 ml/min/1.73m^2^, incident CKD, ≥30% decline in eGFR, and ESRD or ≥50% decline in eGFR, respectively (Table 2 and Fig. 2). Results were consistent when exposure was alternatively assessed using National Aeronautics and Space Administration (NASA) data, where the proportion of the association mediated by diabetes was 5.8% (95%CI: 5.4–6.3%), 6.2% (5.7–6.9%), 7.7% (7.1–8.4%), and 14.6% (13.1–16.2%) for incident eGFR <60 ml/min/1.73m^2^, incident CKD, ≥30% decline in eGFR, and ESRD or ≥50% decline in eGFR, respectively (Table 3). Mediation results were consistent in sensitivity analyses including: (a) when the exposure was assigned by the nearest air monitoring station within 30 miles (Table 3); (b) when models were additionally adjusted for contextual county level characteristics (Table 3); (c) when accelerated failure time models were used (Table 3); and (d) when the non-composite outcome of ≥50% decline in eGFR was assessed, where we observed a proportion mediated of 16.9% (12.9–19.3%).

**Table 3:** Sensitivity analyses of the proportion of the association between PM_2.5_ and kidney disease mediated by diabetes.

Analytic Model	PM_2.5_ Exposure Source	Proportion Mediated (%) (95% CI)			
		Incident eGFR Less Than 60 ml/min/1.73 m^2^	Incident CKD	≥30% Decline in eGFR	ESRD or ≥50% Decline in eGFR
Cox	NASA	5.8 (5.4–6.3)	6.2 (5.7–6.9)	7.7 (7.1–8.4)	14.6 (13.1–16.2)
Cox^+^	EPA^+^	4.3 (3.3–5.0)	4.5 (3.5–5.3)	6.4 (4.9–7.6)	19.8 (13.9–25.2)
Cox*	EPA	4.6 (3.9–5.6)	4.6 (4.1–5.5)	6.0 (4.9–7.2)	11.9 (8.9–13.6)
Accelerated Failure Time	EPA	4.6 (4.2–5.7)	4.8 (4.3–5.9)	6.2 (5.4–7.3)	16.9 (13.1–20.3)

Models adjusted for age, race, gender, cancer, cardiovascular disease, chronic lung disease, hyperlipidemia, T_0_ eGFR, body mass index, smoking status, ACEI/ARB use, systolic blood pressure, diastolic blood pressure, number of outpatient eGFR measurements, number of hospitalizations, county population density, and county percent in poverty.

^+^Nearest air monitoring station within 30 miles of a participant’s residence.

*Model additionally adjusted for contextual county characteristics (n = 1,785,657).

Abbreviations: PM_2.5_, particulate matter <2.5 µm in aerodynamic diameter; CI, 95% confidence interval; eGFR, estimated glomerular filtration rate; CKD, Chronic Kidney Disease; ESRD, end stage renal disease; NASA, National Aeronautics and Space Administration.

## Discussion

While PM_2.5_ is associated with diabetes, a causal driver of CKD, our findings suggest it mediates a small proportion of the association of PM_2.5_ and risk of kidney outcomes. The corollary observation is that a significant proportion of the association between PM_2.5_ and kidney outcomes may reflect a direct relationship. The findings will likely inform more accurate estimation of the burden of kidney disease attributable to air pollution — an issue of rising importance on the global health agenda.

There is increasing realization that pollution is a major driver of non-communicable diseases around the globe^8^ where the majority (71%) of the 9 million annual deaths attributed to pollution are caused by non-communicable diseases. The Lancet Commission on Pollution and Health and subsequent reports specifically outlined the need to define and estimate the burdens of diabetes and kidney disease attributable to air pollution^9–11^. The United Nations high level meeting on non-communicable diseases, held in September 2018, outlined a shift in framework from the four-by-four to the five-by-five approach and added environmental risk factors as key drivers of non-communicable diseases^11^. The World Health Organization now formally recognizes environmental air pollution as a risk factor for non-communicable diseases^11^. Accurate estimation of the burden of non-communicable diseases (including diabetes and kidney disease) is critically important to help inform this effort. The Global Burden of Disease study estimated that 82 million disability-adjusted life-years, a measure of the number of years of healthy life lost, are attributable to PM_2.5_ in 2016, with 10.1 million due to diabetes^12^. Disability-adjusted life-years due to CKD attributable to PM_2.5_ globally in 2016 has been estimated to be 11.5 million^13^. Our results provides a quantitative estimation of the portion of relationship between PM_2.5_ and kidney disease that is mediated by diabetes, and suggests that burdens of diabetes and CKD attributable to PM_2.5_ may only marginally overlap.

The biologic mechanism of a direct relationship between PM_2.5_ and kidney disease is not entirely clear. Prior experimental and human evidence show that inhaled nano-particles, when sufficiently small, enter the bloodstream and are excreted in the urine in animals and humans^14^, suggesting a putative size-dependent pathway where inhaled particles may get in contact with kidney tissue to exert their pathologic effect. Experimental evidence in mice and rats suggests that inhalation of PM_2.5_ leads to significant structural kidney glomerular and tubular changes including tubular atrophy, mesangial expansion, advanced glomerulosclerosis, and decreased glomerular and tubular lumen volumes^15,16^. Experimental findings from Nemmar and collaborators suggest that exposure to diesel exhaust particles (which experimentally simulate environmental exposure to PM_2.5_) leads to disturbances of kidney hemodynamics (and alteration of kidney blood flow) and kidney vascular damage, promotes oxidative stress, inflammation, and DNA damage in kidney tissue, exacerbates acute kidney injury, and further promulgates chronic kidney injury in murine models^17–19^. Further research is needed to further define the mechanistic pathway(s) in which PM_2.5_ adversely affect kidney function and contributes to the biology of CKD.

The magnitude of the proportion mediated by diabetes was higher for the outcome of ESRD or ≥50% decline in eGFR than other kidney outcomes (Table 1). This is most likely a reflection of the strength of the association between diabetes and ESRD relative to the other kidney disease outcomes; CKD due to diabetes progresses more rapidly, and as such it is more likely to lead to a severe and terminal outcome (such as ESRD).

The study has several limitations. The cohort consisted of United States veterans; results may not be generalizable to other populations. Diabetes and PM_2.5_ were assessed concurrently, and therefore temporality could not be established. Our analyses did not consider the source or chemical composition and toxic content of PM_2.5_, which may exhibit regional variation, however, studies have shown that estimates using non-specific PM_2·5_ biomass alone will underestimate the burden of disease attributable to PM_2.5_ pollution^20^. In our analyses we used population level exposure estimates rather than individual measurements, which may have resulted in exposure misclassification. Our datasets did not include data on other air pollutants (ambient coarse particulate matter of ≤10 μm in aerodynamic diameter, nitrogen dioxide, and carbon monoxide, and others), and other parameters including temperature, and humidity, and analyses did not account for potential geographic heterogeneity in effect, which may have biased results^21^. Although we took careful measures to account for potential confounders, and tested a negative exposure control to address potential spurious associations, and we conducted a sensitivity analysis adjusting for contextual county level characteristics as a means of investigating whether shared contextual confounders influenced results, we cannot completely eliminate the possibility that proportion mediated is influenced by residual confounding. Strengths included the use of a large national cohort of US veterans who receive care in a single integrated healthcare network, the use of a negative control to investigate presence of possible hidden bias, and consistency of results across analyses using different data sources to define exposure.

In summary, our results suggest that a small proportion of risk of kidney disease outcomes associated with PM_2.5_ exposure is mediated by diabetes. A greater understanding of mechanisms underlying the direct relationship between PM_2.5_ pollution and risk of kidney disease is needed.

## Methods

### Cohort participants

**Figure 3:**
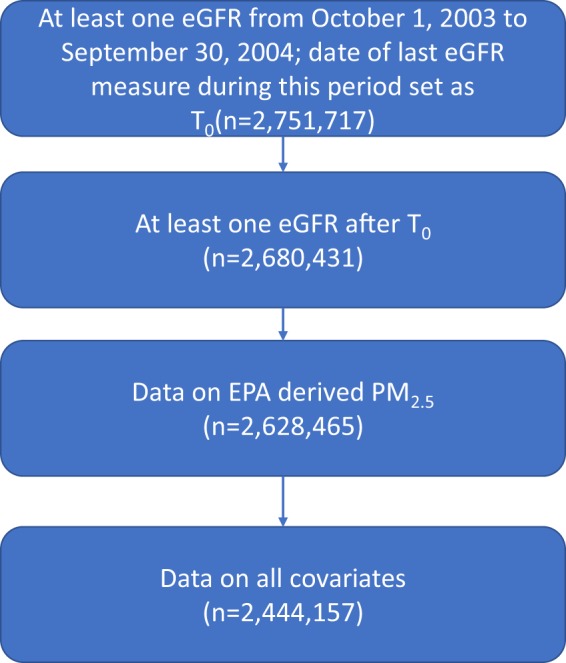
Cohort flow chart.

United States Department of Veterans Affairs (VA) Healthcare System users with a minimum of one outpatient eGFR measurement between October 1, 2003 and September 30, 2004 and no previous history of ESRD were selected from VA data, where the date of last eGFR measurement during this time period was designated time zero (T_0_) (n = 2,751,717). Participants were additionally selected based on the criteria of having at least one outpatient eGFR measurement after T_0_ (n = 2,680,431), and were followed until September 30, 2012 or death. The final analytic cohort was subsequently chosen by restriction to those who had Environmental Protection Agency (EPA) derived PM_2.5_ (n = 2,628,465) data and data on all covariates, yielding a final analytic cohort of 2,444,157 (Fig. 3). This study was approved by the Institutional Review Board of the VA Saint Louis Health Care System, Saint Louis, MO. The study was carried out in accordance with relevant guidelines and regulations. A waiver of informed consent was granted by the Institutional Review Board of the VA Saint Louis Health Care System.

### Data sources

Participant’s demographics, inpatient and outpatient data, laboratory information, vital signs, and medications were obtained from Department of Veterans Affairs datasets, which is collected through routine care received at the VA Health Care System^22–28^. Further details on VA datasets are provided in the Supplementary Methods. Data from the United States Renal Database System (USRDS) obtained through the VA/Centers for Medicare and Medicaid Services (CMS) was utilized in assessing ESRD status^29^. The Center for Disease Control and Prevention’s (CDC) National Environmental Public Health Tracking Network provided annual particulate matter estimates for the contiguous United States that originate from Community Multiscale Air Quality (CMAQ) modeled output^30,31^, which is based on United States Environmental Protection Agency’s (EPA) Air Quality System (AQS) data. EPA data also provided information on ambient air sodium levels, as well as the latitude and longitude of the EPA air monitoring stations whose measures were obtained^32^. NASA Socioeconomic Data and Applications Center Global Annual PM_2.5_ Grids from Moderate Resolution Imaging Spectroradiometer, Multi-angle Imaging Spectro Radiometer and Sea-Viewing Wide Field-of-View Sensor Aerosol Optical Depth with Geographically Weighted Regression (version 1) remote space-borne satellite sensing data served as an additional source of ambient PM_2.5_ estimates at the 10 × 10 km resolution^33,34^. The Census Bureau’s Model-based Small Area Income & Poverty Estimates (SAIPE) supplied annual estimates of county level percent in poverty^35^. Information on county level population density was obtained from the 2000 Census of Population and Housing^35^. Latitude and longitude for ZIP code tabulation areas, which are used in place of ZIP codes in order to define a concrete geographic area, was obtained from the 2000 Census Gazetteer File^36^. Contextual county-level characteristics were curated from the County Health Rankings datasets and encompassed data in several domains including demographics, physical environment, social and economic factors, and health behaviors^37^. Further information on the county level characteristics may be found in the Supplementary Methods.

### Outcomes

Outcomes assessed were time until incident eGFR <60 ml/min/1.73 m^2^, incident CKD (defined as two eGFR <60 ml/min/1.73 m^2^ at least 90 days apart^23^), greater than or equal to 30% decline in eGFR from eGFR at T_0_, and the composite outcome of ESRD or greater than or equal to 50% decline in eGFR^38^. Incident eGFR <60 ml/min/1.73 m^2^ and incident CKD were assessed in sub-cohorts who had no prior history of these outcomes, n = 1,679,965 and 1,616,153 respectively. Patients were censored after onset of ESRD (for non-ESRD outcomes) and at time of death or end of study follow-up. ESRD was ascertained through linkage of USRDS and VA databases. Outpatient eGFR was used in the evaluation of all outcomes except for ESRD. ESRD and greater than or equal to 50% decline in eGFR were combined into a composite outcome due to the low event rate of ESRD. eGFR was calculated using the four-variable abbreviated Chronic Kidney Disease Epidemiology Collaboration (CKD-EPI equation) on the basis of age, race, gender, and serum creatinine^39^.

### Exposure

Baseline county of residence^40^ was linked with ambient fine particulate matter air pollutant exposure data from the EPA CMAQ modeled output^31^. We also linked zip code of residence with exposure data from NASA as an alternative exposure source^34^. Further details on NASA exposure assignment may be found in the Supplementary Methods. Diabetic status was jointly defined by the presence of International Classification of Diseases (ICD-9) codes and diabetic medication, including oral glycemic agents and insulin, in the year prior to, and including T_0_, as a three-level variable: no diabetes, diabetes diagnosis but no medication use, and diabetic medication use.

### Covariates

Baseline covariates were ascertained from October 1, 1999 until cohort entry (T_0_). Covariate selection was informed by prior knowledge^1,2,4,41^; covariates were chosen based on potential confounding of the association of PM_2.5_ with diabetes and adverse kidney outcomes, and of the diabetes and adverse kidney outcomes association. Covariates included age, race, gender, cancer, cardiovascular disease, chronic lung disease, hyperlipidemia, hypertension, T_0_ eGFR, systolic blood pressure, diastolic blood pressure, body mass index, smoking status, angiotensin-converting enzyme inhibitor/angiotensin receptor blocker (ACEI/ARB) use, number of outpatient eGFR measurements, number of hospitalizations, county population density, and county percent in poverty. Covariates were treated as continuous variables where appropriate unless otherwise indicated. Race/ethnicity was categorized as white, black, or other (Latino, Asian, Native American, or other racial/ethnic minority groups). Comorbidities were assigned on the basis of relevant ICD-9-CM diagnostic and procedure codes and Current Procedural Terminology (CPT) codes in the VA Medical SAS datasets using definitions validated for use in VA datasets^1,2,25–27,42–47^. The values for systolic and diastolic blood pressure consisted of the average of all measures in the year prior to T_0_. Body mass index was modeled as a restricted cubic spline. Smoking status was defined as current, former, or never smoker. ACEI/ARB use was defined as use if there were prescriptions for 90 days or greater during the time before T_0_. Number of outpatient eGFR measurements represented the cumulative number of outpatient eGFR values from October 1, 1999 to T_0_. Number of hospitalizations was derived from the cumulative number of inpatient stays lasting a full day or longer from October 1, 1999 to T_0_. Population density and percent in poverty were assigned based on county of residence at T_0_.

### Statistics

Descriptive statistics are presented as frequency (percent) and mean (standard deviation) or median (interquartile range). Adjusted incident rates, per 100,000 persons, of the adverse kidney disease outcomes were calculated by PM_2.5_ quartile using a Poisson regression applied to individual level data; rates were adjusted by age, race, sex, and T_0_ eGFR. Adjusted multinomial logistic generalized estimation equations were used in estimating the association between PM_2.5_ and diabetes, and Cox proportional hazard models with a robust sandwich variance estimator were utilized in estimating associations between PM_2.5_ and time until CKD outcomes, and diabetes and time until CKD outcomes. Mediation analyses were conducted using the inverse odds ratio-weighting (IORW) method^48,49^. Briefly, the IORW method first regresses the exposure, as the outcome in a model, on all mediators and covariates. Results from this model are used to generate weights; results from a weighted and unweighted models provide measures of the direct and total effect, respectively, from which the indirect effect (the proportion mediated) may be calculated. Further details on the inverse odds ratio-weighting method may be found in the Supplementary Methods. Linearity of terms was assessed through restricted cubic spline plots and a Wald chi-squared test for non-linearity. Variables that deviated from linearity were transformed or treated as a restricted cubic spline, determined by minimization of model Akaike information criteria (AIC). PM_2.5_ and risk of kidney disease outcomes did not show evidence of strong deviation from linearity, so no spline was used. We repeated the mediation analyses using PM_2.5_ exposure values derived from NASA satellites as an alternative source for exposure data.

The application of negative control in clinical epidemiology studies serves to identify and resolve sources of spurious casual inference^7^. Measurement of air sodium concentrations occur in the same contextual setting as PM_2.5_; and there is no biologic basis to support an association of air sodium concentration with either diabetes, or kidney disease outcomes^1,2,4^. We therefore a) tested the association between ambient air sodium levels and the risk of diabetes, and b) tested the association between ambient air sodium concentrations and risk of kidney outcomes. Missing data was not imputed. In all analyses, a p-values less than 0.05 or a 95% confidence interval containing unity was considered statistically significant. All analyses were conducted in SAS Enterprise Guide 7.1 (SAS Institute, Cary NC).

To test the robustness of study findings we undertook a number of sensitivity analyses: (1) An alternate exposure definition using the air monitoring station nearest a participant’s place of residence, within a maximum 30 miles, was used. (2) Models were additionally adjusted for county level characteristics in domains including demographics, physical environment, social and economic factors, and health behaviors as a means of investigating whether shared regional confounders influenced results^37^; in this analysis, we conducted a principal component analysis to address multicollinearity amongst the different contextual county characteristics, and then selected components with an eigenvalue greater than 1 for inclusion in our models^50^. (3) It has been suggested that when an outcome is not rare, mediation assessed using a proportional hazard model such as the Cox model may be biased^51^; as such, accelerated failure time models with a Weibull distribution, selected based on form of the log negative log plot and best fit via the AIC criteria, were utilized. (4) We analyzed the outcome of greater than or equal to 50% decline in eGFR alone to investigate whether removal of the ESRD (from the composite outcome of eGFR decline >50% and ESRD) outcome modified the results.

## Supplementary information

Supplemental File.

## Data Availability

Data are available through the US Department of Veterans Affairs.
