# Supplementary material for: Diabetes Minimally Mediated the Association Between PM2.5 Air Pollution and Kidney Outcomes
Source: Sci Rep. 2020 Mar 12;10:4586. doi: 10.1038/s41598-020-61115-x (PMC7067761; doi:10.1038/s41598-020-61115-x)
Supplement: Supplementary file 1 — Supplemental File. [file 41598_2020_61115_MOESM1_ESM.docx]

**Supplementary Information**

**Diabetes Minimally Mediated the Association Between PM_2.5_ Air Pollution and Kidney Outcomes**

**Benjamin Bowe**^1,2,3^**, Yan Xie**^1,2,3^**, Yan Yan**^1,4^**, Hong Xian**^1,2^**, and Ziyad Al-Aly**^1,3,5,6,7*^

^1^Clinical Epidemiology Center, Research and Education Service, VA Saint Louis Health Care System, Saint Louis, Missouri

^2^Department of Epidemiology and Biostatistics, College for Public Health and Social Justice, Saint Louis University, Saint Louis, Missouri

^3^Veterans Research & Education Foundation of St. Louis, Saint Louis, Missouri

^4^Division of Public Health Sciences, Department of Surgery, Washington University School of Medicine, Saint Louis, Missouri

^5^Department of Medicine, Washington University School of Medicine, Saint Louis, Missouri

^6^Nephrology Section, Medicine Service, VA Saint Louis Health Care System, Saint Louis, Missouri

^7^Institute for Public Health, Washington University in Saint Louis, Saint Louis, Missouri

**Corresponding Author*:**

Ziyad Al-Aly, M.D.

VA Saint Louis Health Care System

915 North Grand Boulevard, 151-JC

Saint Louis, MO 63106

Telephone: (314) 289-6333

Twitter [@zalaly](https://twitter.com/zalaly/)

email: [zalaly@gmail.com](mailto:zalaly@gmail.com)

**Table of Contents:**

| **Item** | **Pages** |
| --- | --- |
| **Supplementary Methods** | |
| VA data sources | 3 |
| NASA PM_2.5_ exposure assessment | 4 |
| Mediation analyses: Inverse odds ratio-weighting | 5 |
| References | 6 |
| **Supplementary Tables** | |
| **Supplementary Table S1:** Adjusted incident rates per 100,000  person years (95% CI) by PM_2.5_ Quartile. | 7 |
| **Supplementary Table S2:** Association of ambient air sodium  with kidney disease outcome and diabetes. | 8 |
| **Supplementary Figures** | |
| **Supplementary Figure S1:** Geographic distribution of cohort participants. | 9 |
|  |  |

**VA Data Sources**

VA datasets including inpatient and outpatient medical SAS datasets that contain utilization data, participant data on location based on Federal Information Processing Standard (FIPS) county codes and ZIP codes, demographic characteristics, and comorbidity information based on Current Procedural Terminology (CPT) codes, and International Classification of Diseases, Ninth Revision, Clinical Modification (ICD-9-CM) diagnostic and procedure codes^1-4^. The VA Managerial Cost Accounting Laboratory Results file, which includes results for select laboratory tests obtained in the clinical setting, supplied data on outpatient and inpatient serum creatinine measurements collected during routine medical care^1,2,5^. The VA Vital Status and Beneficiary Identification Records Locator Subsystem (BIRLS) files supplied death follow-up through September 30, 2012, and demographics^1,2^. The Corporate Data Warehouse (CDW) Vital Signs, Health Factors, and RX Outpatient domains provided data on body mass index (BMI), systolic and diastolic blood pressure, smoking status, and angiotensin-converting enzyme inhibitors (ACEIs) / angiotensin receptor blockers (ARBs) and diabetic medication (including oral hypoglycemic agents and insulin) use.

**NASA PM_2.5_ Exposure Assessment**

NASA’s SEDAC Global Annual PM2.5 Grids from MODIS, MISR and SeaWiFS Aerosol Optical Depth (AOD) provided the data on PM_2.5_ estimates at the 10x10 km resolution^6,7^. Data was available as a three-year median of years 2003-2005. Overlap of 10x10 km resolution PM_2.5_ grids and ZIP code’s geographic area, resulting in surface area weighted PM_2.5_ values for each ZIP code, was used to define exposure levels (as depicted in the figure below).


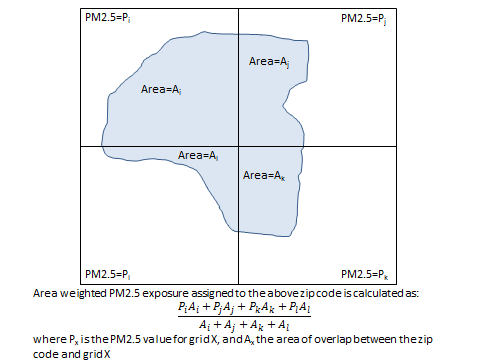


**Mediation analyses**

Mediation analyses were conducted using the inverse odds ratio-weighting method, chosen for its flexibility in assessing mediation by multiple levels of a variable at once and its application to survival models^8,9^. Mediation analyses assume that there is no unmeasured confounding of the effect of the exposure on the mediator, the exposure on the outcome, and the mediator on the outcome. It also assumes that there are no confounding variables of the mediator to outcome relationship that is affected by the exposure^9^. Weighting methods are used to calculate the total effect, or full association of the exposure with the outcome, and the direct effect, or the part of the exposure to outcome association that is not mediated. This can then be used to calculate the indirect effect (using the difference method), or the part of the association that is mediated. The algorithm for this method is^39^:

1. Regress the primary exposure variable, as the outcome, on mediators (and covariates). In the case where the exposure is continuous, a linear regression (1) may be used:

$E=\beta_{0}+\beta_{1}M_{1}+\beta_{2}M_{2}+\beta_{3}^{'}C+e$ (1)

Where *E* is the exposure, $\beta_{0}$ the intercept, $\beta_{1}$ the coefficient for mediator $M_{1},$ $\beta_{2}$ the coefficient for mediator $M_{2},$ $\beta_{3}^{'}$ a vector of coefficients for covariates $C$, and *e* the residuals with normal distribution $N(0,\sigma^{2})$.

1. Use regression coefficients and the variance of the residuals from step 1 to calculate a weight for each participant. In this setting the equation (2) uses:

$\frac{1}{OR(E,M|C)}=\frac{1}{exp(\left[ \left( \beta_{1}*E*M_{1})+\beta_{2}*E*M_{2} \right) \right]/{\sigma^{2}})}$ (2)

1. Regress the outcome of interest on the primary exposure variable *E* and covariates *C* (without mediators). The regression coefficient of the exposure from this model represents the total effect.
2. Regress the outcome of interest on the primary exposure variable *E* while controlling for covariates *C* (without mediators), weighted per step 2. The regression coefficient of the exposure is the direct effect.
3. The indirect effect may be then calculated by subtracting the direct effect from the total effect. The proportion of the indirect / total effect gives the proportion mediated.
4. Bootstrap to derive confidence interval.

**References:**

1 Murphy, P. A., Cowper, D. C., Seppala, G., Stroupe, K. T. & Hynes, D. M. Veterans Health Administration inpatient and outpatient care data: an overview. *Effective clinical practice : ECP* **5**, E4 (2002).

2 Oddone, E. Z. & Eisen, S. Veterans Affairs Research and Development: using science to improve health care for veterans. *North Carolina medical journal* **69**, 35-37 (2008).

3 VIReC Research User Guide: VHA Medical SAS® Outpatient Datasets FY2006. U.S. Department of Veterans Affairs. VA Information Resource Center: Hines, I., September 2007.

4 VIReC Research User Guide: VHA Medical SAS® Inpatient Datasets FY2006. U.S. Department of Veterans Affairs. VA Information Resource Center: Hines, I., September 2007.

5 VIReC Research User Guide: Veterans Health Administration Decision Support System Clinical National Data Extracts, H., IL, U.S. Department of Veterans Affairs. VA Information Resource Center September 2009.

6 van Donkelaar, A., Martin, R. V., Brauer, M. & Boys, B. L. Use of Satellite Observations for Long-term Exposure Assessment of Global Concentrations of Fine Particulate Matter. *Environmental Health Perspectives* **123**, 135-143 (2015).

7 van Donkelaar, A., Martin, R. V., Brauer, M. & Boys, B. L. (NASA Socioeconomic Data and Applications Center (SEDAC), Palisades, NY, 2015).

8 Tchetgen Tchetgen, E. J. Inverse odds ratio‐weighted estimation for causal mediation analysis. *Statistics in medicine* **32**, 4567-4580 (2013).

9 Nguyen, Q. C., Osypuk, T. L., Schmidt, N. M., Glymour, M. M. & Tchetgen Tchetgen, E. J. Practical guidance for conducting mediation analysis with multiple mediators using inverse odds ratio weighting. *American journal of epidemiology* **181**, 349-356 (2015).

| **Supplementary Table S1:** Adjusted incident rates per 100,000 person years (95% CI) by PM_2.5_ Quartile. | | | | |
| --- | --- | --- | --- | --- |
| **Quartile** | **Incident eGFR Less Than 60^+^** | **Incident CKD*** | **≥ 30% Decline in eGFR** | **ESRD or** ≥**50% decline in eGFR** |
| 1 | 7082.7  (7048.0-7117.4) | 3638.5  (3616.9-3660.1) | 4144.6  (4129.1-4160.1) | 1237.3  (1229.6-1245.0) |
| 2 | 7825.5  (7794.4-7856.6) | 4087.3  (4067.7-4106.9) | 4724.0  (4708.2-4739.8) | 1496.2  (1487.5-1504.9) |
| 3 | 8031.0  (7999.1-8062.9) | 4230.7  (4211.9-4249.5) | 4837.1  (4819.4-4854.8) | 1494.7  (1486.9-1502.5) |
| 4 | 8516.2  (8477.1-8555.3) | 4649.1  (4627.2-4671.0) | 5337.6  (5317.4-5357.8) | 1709.2  (1701.1-1717.3) |
| Adjusted for age, race, gender, and T0 eGFR.  + Incident eGFR<60 was evaluated in a subcohort of people with no prior history of eGFR≤60 at time of cohort entry (n=1,679,965).  *Incident CKD was evaluated in a subcohort of people with at least 2 eGFR separated by at least 90 days apart who had no prior history eGFR≤60 at time of cohort entry (n=1,616,153). | | | | |

| **Supplementary Table S2:** Association of ambient sodium levels with risk of kidney outcomes and diabetes (n=1,164,532). | | | | | | |
| --- | --- | --- | --- | --- | --- | --- |
| **Exposure** | **Outcome** | | | | | |
|  | **Incident eGFR Less Than 60**  **HR (CI)** | **Incident CKD**  **HR (CI)** | ≥**30% Decline in eGFR**  **HR (CI)** | **ESRD or** ≥**50% Decline in eGFR**  **HR (CI)** | **Diabetes** | |
|  |  |  |  |  | **ICD-9 only**  **OR (CI)** | **Medication**  **OR (CI)** |
| **Sodium*** | 0.98 (0.98-0.99) | 0.97 (0.97-0.98) | 0.98 (0.98-0.99) | 1.00 (0.99-1.00) | 1.01 (1.00-1.03) | 0.98 (0.96-0.99) |
| Models adjusted for age, race, gender, cancer, cardiovascular disease, chronic lung disease, hyperlipidemia, T_0_ eGFR, body mass index, smoking status, ACEI/ARB use, systolic blood pressure, diastolic blood pressure, number of outpatient eGFR measurements, number of hospitalizations, county population density, and county percent in poverty.  *For every IQR (0.046 µg/m^3^) increase. | | | | | | |

**Supplementary Figure legends:**

**Supplementary Figure S1:** Geographic distribution of cohort participants. States are colored by participant number decile.
